# Intrusion Detection System for IoT Based on Deep Learning and Modified Reptile Search Algorithm

**DOI:** 10.1155/2022/6473507

**Published:** 2022-06-02

**Authors:** Abdelghani Dahou, Mohamed Abd Elaziz, Samia Allaoua Chelloug, Mohammed A. Awadallah, Mohammed Azmi Al-Betar, Mohammed A. A. Al-qaness, Agostino Forestiero

**Affiliations:** ^1^Mathematics and Computer Science Department, University of Ahmed DRAIA, 01000 Adrar, Algeria; ^2^LDDI Laboratory, Faculty of Science and Technology, University of Ahmed DRAIA, 01000 Adrar, Algeria; ^3^Faculty of Science &Engineering, Galala University, Suez, Egypt; ^4^Artificial Intelligence Research Center (AIRC), College of Engineering and Information Technology, Ajman University, Ajman, UAE; ^5^Department of Mathematics, Faculty of Science, Zagazig University, Zagazig 44519, Egypt; ^6^Department of Information Technology, College of Computer and Information Sciences, Princess Nourah bint Abdulrahman University, P.O.Box 84428, Riyadh 11671, Saudi Arabia; ^7^Department of Computer Science, Al-Aqsa University, P.O. Box 4051, Gaza, State of Palestine; ^8^Department of Information Technology, Al-Huson University College, Al-Balqa Applied University, Irbid, Jordan; ^9^State Key Laboratory for Information Engineering in Surveying, Mapping and Remote Sensing, Wuhan University, Wuhan 430079, China; ^10^Institute for High Performance Computing and Networking, National Research Council, Rende(CS), Italy

## Abstract

This study proposes a novel framework to improve intrusion detection system (IDS) performance based on the data collected from the Internet of things (IoT) environments. The developed framework relies on deep learning and metaheuristic (MH) optimization algorithms to perform feature extraction and selection. A simple yet effective convolutional neural network (CNN) is implemented as the core feature extractor of the framework to learn better and more relevant representations of the input data in a lower-dimensional space. A new feature selection mechanism is proposed based on a recently developed MH method, called Reptile Search Algorithm (RSA), which is inspired by the hunting behaviors of the crocodiles. The RSA boosts the IDS system performance by selecting only the most important features (an optimal subset of features) from the extracted features using the CNN model. Several datasets, including KDDCup-99, NSL-KDD, CICIDS-2017, and BoT-IoT, were used to assess the IDS system performance. The proposed framework achieved competitive performance in classification metrics compared to other well-known optimization methods applied for feature selection problems.

## 1. Introduction

The emerging technology of the Internet of Things (IoT) is constantly evolving and being exploited in the last couple of years, enabling communications and interactions among several devices via a network; thus, it is propelling new technology of business process [1]. Subsequently, several challenges in many aspects, such as financially, in proving credibility, in the enforcement, and in business operations, have come to the fore resulting from the exponential growth of cybersecurity attacks [2]. Cloud computing is normally used as an IoT data storage, which is formulated as a model that supplies various resources and services to the customer on-demand. Typically, cloud computing minimizes the human intervention between users and providers [3]. Due to its impressive features, it has received serious attention from organizations and users. However, to transit from the current platform to the cloud computing platform, several struggling issues can be faced related to the operation mechanism and security. The vulnerability of cloud computing is related to the valuable data stored remotely on servers. This security threat makes it a target for many cybercriminals and intruders; therefore, it hinders many people from favoring or transiting to the cloud computing platform. There are several reasons why the recent cyberattacks are substantially growing. One of the main reasons is related to the existence and accessible hacking tools that can be easy to use, which allow the naive hackers to quickly attack the cloud storage without brilliant skills or specific knowledge [4–8].

In the last decades, a considerable inattention from a wide range of research communities has been paid to address different issues in cyberattacks domain such as intrusion detection systems (IDSs) [9]. Furthermore, various machine learning (ML) algorithms were utilized to address the cyberattack issues such as the implementation of the decision tree algorithm (DT) in [10, 11], support vector machine (SVM) models in [12, 13], k-means [14, 15], k-nearest neighbor (kNN) [16, 17], and many other machine learning algorithms [18–20]. Quite recently, many deep neural network solutions have been applied to the IDS in fog, clouds, and other IoT-based systems. Notably, the convolutional neural network (CNN) model [21] and the deep recurrent neural network (RNN) model in [22], as well as the restricted Boltzmann machines (RBMs) in [23], multilayered perceptron neural network [24], and many others [25].

The IDS is modeled as a feature selection problem and has been successfully addressed by various traditional classifiers. As the revolution of metaheuristic (MH) optimization algorithms, they used to tackle a wide range of complex optimization problems. MH is essentially utilized for IDSs such as the particle swarm optimization (PSO) algorithm [26], crow search algorithm (CSA) [27], genetic algorithm [28–30], random harmony search algorithm [31], and grey wolf optimizer (GWO) algorithm [32, 33].

In this article, we propose a novel powerful IDS model utilizing advanced versions of deep learning (DL) and metaheuristic optimization algorithms. The features initially were extracted efficiently and simply by implementing a convolutional neural network (CNN) model. There are many consecutive convolution blocks designed to extract the informative features. The CNN was only used in the feature extraction phase, which allows the extraction of meaningful features that can represent the raw data in a lower-dimensional space. In addition, CNNs are well known for their ability to learn complex features with less complex architectures and fast training processes. Following the blocks in CNN, the fully connected layer is built to extract relative features and detect malicious or intruder activities. Thereafter, a new and efficient version of the reptile search algorithm (RSA) [34] is proposed as a feature selection tool to improve the classification results of IDS. The RSA is used since it is a very recent but efficient algorithm due to several impressive features, such as it has few parameters to be initiated. In the initial search, the derivative information is not mandatory. It is simple and easy to use. It is scalable and admissible. Finally, it is sound and complete. Therefore, it has been tested against several benchmark functions and engineering problems [34]. The RSA also helps in improving the neuro-fuzzy inference system for predicting the swelling potentiality for fine-grained soils[35]. Although the RSA has several advantages, as with other MH algorithms, its performance can be affected by the problem size and complexity. Accordingly, the RSA suffers from premature convergence due to the lack of balance between the exploration and exploitation capabilities during the search. Therefore, the problem-specific knowledge embedded with the search space shall be considered, and a suitable adjustment to the RSA optimization structure shall be adopted.

The designed model proposed in this study was initiated by preparing the IoT dataset for feature extraction. The feature extractor model is a CNN model that is trained over the preprocessed dataset. The outputs of the CNN model, which are the extracted features, are filtered, and the most relevant features are selected by the RSA. To evaluate the proposed model, four public datasets, KDDCup-99, NSL-KDD, Industrial IoT (IIoT) traffic data (BoT-IoT), and CICIDS-2017 were used. Furthermore, the results of the proposed RSA-based model are evaluated against the other seven well-established algorithms. The comparative results demonstrate the viability of our developed model, which shows significant performance for all datasets.

Our main objective of this study is to propose a novel and efficient IDS model that utilizes the impressive features of efficient deep learning and MH algorithms. To achieve these objectives, several contributions are presented in this article as follows.

Design a CNN model as a feature extractor with the goal of extracting the feature from the mentioned IoT datasets. Propose an adapted version of the RSA as a feature selection technique for selecting the most relevant and informative features. Assess the model by comparing its yielded results against seven state-of-art models over five well-known public datasets.

The remaining parts of this article are organized as follows: Section 2 reviews the related research works on IDS models. Then in Section 3, we elaborate on the basics and fundamentals for RSA. The proposed IoT security model is presented in Section 4. The results and discussion is given in Section 5. Finally, in Section 6, the conclusion of the article is stated, and the possible future works are recommended.

## 2. Related Works

The related works of some previous IDS utilizing metaheuristic algorithms are summarized. The deep learning model and swarm intelligence approaches are combined by Saljoughi et al. [36] to address the IDS scheme for cloud computing. The authors used multilayer perceptron (MLP) neural networks as a feature extractor and the particle swarm optimization (PSO) as a feature selection method. Two datasets are used for evaluation purposes: KDD-CUP and NSL-KDD. Their proposed method yielded a significant performance in detecting intruders and cyberattacks through experimental validation.

Also, in [37], the denial-of-service (DOS) attack detection in cloud computing is tackled using an enhanced version of the artificial bee colony metaheuristic, which is utilized for boosting the classifier's performance [37]. Their developed system can achieve prediction results with a 72.4% average detection rate when compared to QPSO. In [38], Dash suggests two IDS methods based on the artificial neural networks algorithm for intrusion detection and metaheuristic algorithms. The first method suggests utilizing the gravitational search (GS) algorithm during the second combined GS with PSO. The two methods (GS and GS-PSO) are used as a trainer for the ANN. Their performance is validated using comparative evaluation against several well-established algorithms such as gradient descent, PSO, and GA.

The literature indicates the significant use of various metaheuristics in line with machine learning classifiers for security protection applications, where the metaheuristic algorithms will be utilized as feature selection optimizers and the classifiers as improper action detectors. For instance, the authors of [39] reported significant outcomes for KDD-CUP 99 datasets, where an intrusion detection system is composed of a genetic algorithm and fuzzy support vector machine (SVM). Similarly, Nazir and Khan built a Tabu Search Random Forest (TS-RF), which is a strong intrusion detection system (IDS) in [40], such that the TS algorithm was integrated with the RF classifier. The performance of the system was tested using the UNSW-NB15 dataset, where the results revealed an improvement in the classification accuracy compared to several other methods.

In addition, an improved intrusion detection system was proposed by Mayuranathan et al. in [31], where the feature selection mechanism was optimized by applying the random harmony search algorithm (RHS) and the distributed DoS (DDoS) detection was performed by implementing the restricted Boltzmann machine classifier. The system was tested utilizing the KDD'99 datasets, and the results denoted a considerable detection performance.

On the other hand, other authors utilize neural network classifiers in their systems. In particular, an intrusion detection system for the Internet of medical things applications [33] was built by integrating the hybrid of principal component analysis (PCA), grey wolf optimizer (GWO) algorithm, and deep neural network (DNN). The PCA-GWO was used to optimize the performance of the classier (DNN). The feature selection optimization was reflected in the results and indicated a respective classification accuracy.

Furthermore, a new denial-of-service (DoS) detection system was proposed in [27] by SaiSindhuTheja and Shyam. The system optimizes the feature selection mechanism with the use of the modified crow search algorithm (CSA), such that for optimization performance enhancement, integration between the crow search algorithm (CSA) and the opposition-based learning (OBL) is implemented. Consequently, the second component of the system is the classifier, where the recurrent neural network (RNN) will be utilized for this task. The strength of the system gave it the ability to compete and outperform other detection systems.

## 3. Background

This section provides the two main aspects of the RSA as follows: the inspirations of the RSA are illustrated in Section 3.2, while the detailed descriptions of the procedural steps of RSA are shown in Section 3.3. In addition, this section presents a brief introduction to CNN-based models and their applications in the following section.

### 3.1. Convolutional Neural Network

Nowadays, AI-based algorithms such as CNNs have been widely exploited in fields such as computer vision. For instance, CNNs were extensively used to identify the COVID-19 and quickly diagnose image data. This section will briefly cover the recent advances and existing literature on using CNNs in different applications. Depending on the CNN architecture and building blocks, the CNN models can be applied to various data, including time-series data, textual data, images, and videos [41]. Thus, the main crucial component of such a model is the convolution operation applied to the input data. The convolution operation extracts features from the input data using several convolutional filters with the same or different filter sizes. In addition, the convolution operation relies on the local correlation of the information, which can help extract more complex features and learn more meaningful feature representations. The CNNs can suffer from variations in the data, such as image data (translation, rotation, and scaling). Thus, the CNNs use a pooling operation to sample the feature map extracted from the previous layer. Depending on the task, fully connected (FC) layers can be placed after a convolution block (convolution and pooling) or at the end of the network to classify or detect the input data.

Several CNN architectures have been proposed based on several criterions such as the network depth or width, the type of the convolution operation, the number of convolutional filters and their corresponding size, the pooling operation and its size, the number of fully connected layers, and the deployment environment of the model. Many CNN-based models have been proposed including MobileNet, ResNet, NASNet, EfficientNet, MnasNet, and AlexNet [42–46]. For instance, MobileNet has three versions where MobileNetV3 implements the inverted residual block inherited from EfficientNet and ResNet [47]. The MobileNetV3 uses different types of convolution layers named the depthwise separable convolution, which was proposed to replace the standard convolution operation and lower the computation cost, facilitating the model deployment in embedded and edge systems. In addition, the proposed MobileNetV3 consists of a novel building block named Squeeze-And-Excite block [44]. The depthwise separable convolution uses the inverted residual connection to reduce the number of training parameters and improve the learned representations from the input data. The architectures mentioned above have been employed in a variety of tasks related to computer vision, such as image recognition, classification, image segmentation, face detection, and video classification [48]. The CNNs have shown a great ability in extracting features automatically, even when using simple networks. Thus, in our study, we propose a simple yet effective CNN architecture and adapt it to the network intrusion detection task.

### 3.2. Inspiration of RSA

The RSA is a recently developed metaheuristic algorithm by Abualigah et al. in 2021 [34]. The RSA mimics the hunting behavior of crocodiles in their natural habitat. In general, the crocodiles are belonged to the family of “Crocodylinae,” while they prefer to live in an environment where water and food are available. They are from the amphibians capable of hunting in the water, as well as out of the water. The living behaviors of crocodiles are illustrated as follows:

- Vision: Crocodiles have a penetrating night vision that many other animals lack. They use the disadvantages of most other animals of poor night vision for hunting at night.
- Eating: Crocodiles are predators residing at the top of the food chain, as they are fed from the environment surrounding their habitat such as fishes and deer, cows, zebras, baby elephants, and small crocodiles. In addition, large crocodiles are not afraid to add other predators to their food sources, such as sharks and cats. It also has the ability to live for long periods without food if the surrounding environment lacks any food source. It was reported from the sources that some of them can feed on fruits.
- Locomotion: Crocodiles have the ability to swim, walk, and run. In swimming, they use the tail for steering, and the legs are ignored. In walking, they use their legs to carry their bodies and facilitate their movement, and the tail is used for balancing and steering. Finally, crocodiles can run short distances out of the water to attack prey, and thus, the energy is transmitted from the tail to the body to move forward at high speed.
- Cognition: Crocodiles have the ability to recognize the patterns of prey; for example, they have the ability to know which animals come to water in order to drink frequently.
- Hunting: Crocodiles are set ambushes inside the water to catch animals that come to drink from the water's edge or that dive in the water. At the right moment, crocodiles stealthily attack their prey from the water. Once the crocodile catches its prey, it drags it into the water and drowns it. Finally, the crocodile cuts its prey into large pieces and devours it completely. Frequently, crocodiles fight each other in order to share prey.
- Cooperation: Crocodiles are animals that prefer to live in groups. This pattern helps crocodiles cooperate in order to prepare for ambushes of predation. Everyone in the group has a role in helping accomplish the task of predation. For example, a crocodile attacks the animal that drinks from the riverbank in order to push him towards the water and then the crocodiles hiding in the water attack the prey.

### 3.3. Procedural Steps of RSA

**Figure 1:**
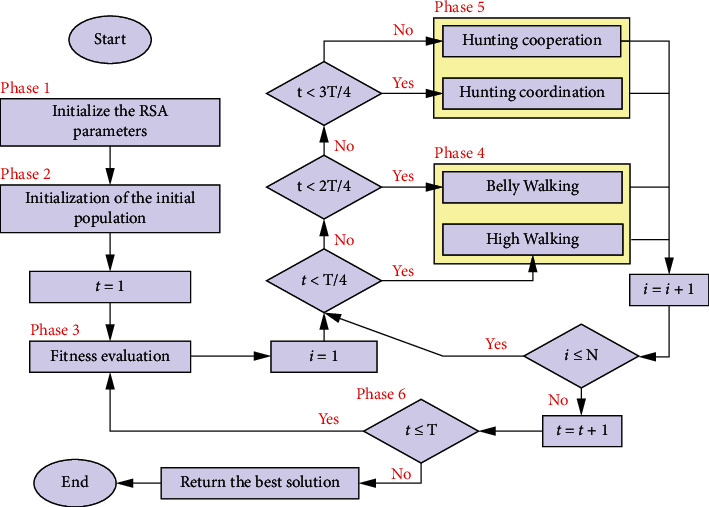
The flowchart of the RSA.

Figure 1 illustrates the procedure steps of the RSA, while a detailed description of these steps is shown.

#### 3.3.1. Phase 1: Initialization of RSA's Parameters

The control parameters and the algorithmic parameters should be initialized before executing the RSA. The list of control parameters includes (*N*), which represents the number of crocodiles, and (*T*) as the maximum number of iterations. Furthermore, two algorithmic parameters are used in RSA, such as *α* and *β*. These two algorithmic parameters are used to control exploitation and exploration abilities, respectively, in order to reach the right balance between the two abilities during the search process.

#### 3.3.2. Phase 2: RSA Population Initialization

During this phase, we randomly generate a set of initial solutions using the following equation [34]:

(1) $$\begin{array}{c} X_{i,j}=X_{j}^{\min}+rn\;d\times \left(X_{j}^{\max}-X_{j}^{\min}\right),\;\forall i=1,2,\ldots ,N,\\ \forall j=1,2,\ldots ,d, \end{array}$$

where *X*_*i*,*j*_ represents the decision variable of the *i* th solution at the *j* th position. The upper and the lower bounds of the decision variable at the *j* th position are *X*_*j*_^max^ and _*j*_*X*^min^. rand is a randomly generated value between 0 and 1, while d indicates the total number of decision variables at each solution. The set of solutions, as many as *N*, are generated and stored in *X* as follows [34]:

(2) $$\begin{array}{c} X=\left[\begin{array}{ccccc} X_{1,1} & X_{1,2} & \ldots & X_{1,d-1} & X_{1,d}\\ X_{2,1} & X_{2,2} & \ldots & X_{2,d-1} & X_{2,d}\\ \vdots & \vdots & \ldots & \vdots & \vdots \\ X_{N,1} & X_{N,2} & \ldots & X_{N,d-1} & X_{N,d} \end{array}\right], \end{array}$$

where each row *X*_*i*_=(*X*_*i*,1_, *X*_*i*,2_,…, *X*_*i*,*d*−1_, *X*_*i*,*d*_) indicates the solution of *i* th position.

#### 3.3.3. Phase 3: Fitness Evaluation

The fitness value (i.e., quality) of each solution in the population should be calculated as *f*(*X*_*i*_) ∀ *i*=1,2,…, *N*.

#### 3.3.4. Phase 4: Encircling Phase

This is the exploration behavior of crocodiles in the RSA. This phase is introduced to find a better solution by exploring new regions in the search space of the problem following two strategies, namely, the high walking and belly walking, as shown in (3). The high walking strategy is controlled by *t* ≤ *T*/4, while the belly walking strategy is controlled by *T*/4 < *t* ≤ 2*T*/4 [34]:

(3) $$\begin{array}{c} X_{i,j}\left(t+1\right)=\left\{\begin{array}{cc} X_{j}^{\text{Best}}\left(t\right)-\eta_{i,j}\left(t\right)\times \beta -R_{i,j}\left(t\right)\times rn\;d, & t\leq \frac{T}{4},\\ X_{j}^{\text{Best}}\left(t\right)\times X_{r1,j}\left(t\right)\times \text{ES}\left(t\right)\times rn\;d, & \frac{T}{4}<t\leq \frac{2T}{4}, \end{array}\right. \end{array}$$

where *X*_*i*,*j*_ represents the decision variable of the *i* th solution at the *j* th position. *X*_*j*_^Best^(*t*) is the *j* th position in the best solution obtained at *t* iteration. *t*+1 is the new iteration, and while the previous iteration is *t*. The hunting operator of the *j* th position in the *i* th solution is denoted as *η*_*i*,*j*_(*t*), which is calculated using (4). The parameter *β* controls the exploration capability of the high walking strategy. The value of *β* is set to 0.1 according to [34]. *rn*  *d* is a randomly generated value ranging between zero and one. *X*_*r*1,*j*_(*t*) is the decision variable at the *j* th position in the *r*1 th solution, where *r*1 ∈ [1, *N*]. *η*_*i*,*j*_(*t*), *P*_*i*,*j*_, and Avg(*X*_*i*_) are calculated, respectively, as follows:

(4) $$\begin{array}{c} \eta_{i,j}=X_{j}^{\text{Best}}\times P_{i,j}, \end{array}$$

(5) $$\begin{array}{c} P_{i,j}=\alpha +\frac{X_{i,j}-\text{Avg}\left(X_{i}\right)}{X_{j}^{\text{Best}}\times \left(X_{j}^{\max}-X_{j}^{\min}\right)+\epsilon }, \end{array}$$

(6) $$\begin{array}{c} \text{Avg}\left(X_{i}\right)=\frac{1}{d}\sum_{j=1}^{d}X_{i,j}, \end{array}$$

where *P*_*i*,*j*_ is the percentage difference between the decision variable at the *j* th position of the best solution *X*^Best^ and the decision variable at same position of the current solution *X*_*i*_. *α* is set to 0.1 according to [34], which is also used to control the exploration ability of the RSA during the hunting cooperation. *ϵ* is a random value between 0 and 2. Avg(*X*_*i*_) is the average value of all decision variables of the current solution *X*_*i*_. *R*_*i*,*j*_(*t*) is a factor used to reduce the search area of the *j* th position in the *i* th solution and ES(*t*) is the evolutionary sense probability and assigns a randomly decreasing value from 2 to -2 [34], which are calculated, respectively, as follows:

(7) $$\begin{array}{c} R_{i,j}=\frac{X_{j}^{\text{Best}}-X_{r2,j}}{X_{j}^{\text{Best}}+\epsilon }, \end{array}$$

(8) $$\begin{array}{c} \text{ES}\left(t\right)=2\times r3\times \left(1-\frac{1}{T}\right), \end{array}$$

where in the equation, *r*2 is a randomly generated value ranging between 1 and *N*, which refers to the index of one solution in the population that is randomly chosen. *r*3 is a random integer value between 0, or 1, or -1.

#### 3.3.5. Phase 5: Hunting Phase

This is the exploitation behavior of crocodiles in the RSA. This phase is designed in the RSA to exploit the current research regions in order to find the optimal solutions according to two strategies: hunting coordination and hunting cooperation, as shown in (9). The hunting coordination strategy is controlled by *t* ≤ 3*T*/4, while the hunting cooperation is controlled by [34].

(9) $$\begin{array}{c} X_{i,j}\left(t+1\right)=\left\{\begin{array}{cc} X_{j}^{\text{Best}}\left(t\right)\times P_{i,j}\left(t\right)\times rn\;d, & \frac{2T}{4}<t\leq \frac{3T}{4},\\ X_{j}^{\text{Best}}\left(t\right)-\eta_{i,j}\left(t\right)\times \epsilon -R_{i,j}\left(t\right)\times rn\;d, & \frac{3T}{4}<t\leq T. \end{array}\right. \end{array}$$

#### 3.3.6. Phase 6: Stop Criterion

Repeat from Step 3 to Step 5 until we reach the maximum number of iterations *T*.

## 4. Proposed Model

**Figure 2:**
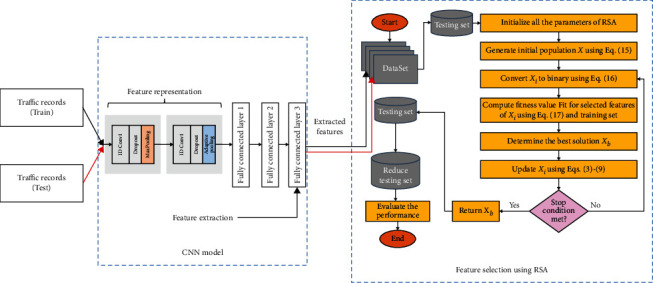
Steps of the presented IoT security method.

With this part, the phases of the proposed IoT security are based on extracting the feature from the data using CNN and then selecting the relevant feature using a modified RSA. In general, the IoT security model consists of four stages, as given in Figure 2 and the description of each phase is given as follows.

### 4.1. First Phase: Prepare IoT Dataset

In this stage, the IoT dataset is prepared to make it suitable for the feature extraction stage (next one). This is achieved by normalizing the dataset using min − max approach. For clarity, by considering the collected traffic samples, TS of IoT is represented as [34]

(10) $$\begin{array}{c} \text{TS}=\left[\begin{array}{cccc} tf_{11} & tf_{12} & \ldots & tf_{1d}\\ tf_{21} & tf_{22} & \ldots & tf_{2d}\\ \ldots & \ldots & \ldots & \ldots \\ tf_{n1} & tf_{n2} & \ldots & tf_{nd} \end{array}\right]. \end{array}$$

Using the min − max approach to normalized TS, DN_ij_ is formulated as [34]

(11) $$\begin{array}{c} {\text{DN}}_{ij}=\frac{tf_{ij}-\min\left({\text{TS}}_{j}\right)}{\max\left({\text{TS}}_{j}\right)-\min\left({\text{TS}}_{j}\right)}, \end{array}$$

where *tf*_*ij*_ indicates the value of feature *j* at the sample *i*. So, the normalization of TS is represented as

(12) $$\begin{array}{c} \text{NTS}=\left[\begin{array}{cccc} {\text{DN}}_{11} & {\text{DN}}_{12} & \ldots & {\text{DN}}_{1d}\\ {\text{DN}}_{21} & {\text{DN}}_{22} & \ldots & {\text{DN}}_{2d}\\ \ldots & \ldots & \ldots & \ldots \\ {\text{DN}}_{n1} & {\text{DN}}_{n2} & \ldots & {\text{DN}}_{nd} \end{array}\right], \end{array}$$

where TS_*i*_ stands for the features of *i* th traffic, and they are represented as [*tf*_11_, *tf*_12_*m*,…, *tf*_1*d*_] of *i*. *n* is the number of samples, and *d* stands for the number of features.

### 4.2. Second Phase: CNN for Feature Extraction

**Figure 3:**
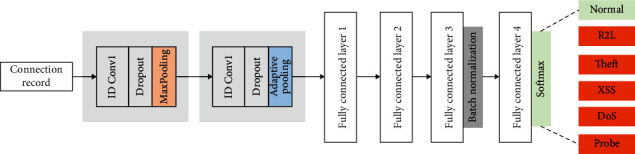
Presented structure of the feature extraction based on CNN.

The CNN is a widely used automatic feature extractor in various applications [49, 50] such as image classification, text classification, speech recognition, and others. In our study, we implemented a CNN model using the following architecture: Input⟶[(Conv)⟶(Pool)] × 2⟶[(FC − 128)] × 2⟶[(FC − 64)⟶(BN − 64)] × 2. The core building blocks are convolution layer (Conv), ReLU activation function, fully connected layer (FC), and pooling layer (Pool). The CNN learns complex representations as features from the network traffic samples and classifies them based on their intrusion type. Using a convolution operation, the CNN extracts local and position-invariant patterns while sharing the weights across the layers and channels [51]. In our case, the design of the CNN network was based on the error, and trial method, where the objective is to build a simple yet powerful model that maximizes the classification accuracy on the tackled task. In addition, the best-trained model based on its performance on the test data is used to extract the learned features for the feature selection stage. The proposed CNN is illustrated in Figure 3.

In the implemented CNN architecture, the Conv block is followed by a rectified linear unit (ReLU) [52] defined in (13) to prevent the negative/small values from being propagated, while the pooling operator is used for reducing the dimensionality of the activation map ReLU(*x*) of the inputted data *x*:

(13) $$\begin{array}{c} \text{ReLU}\left(x\right)=\max\left(0,x\right). \end{array}$$

To reduce the model complexity and prevent overfitting, dropout layers are used with a regularization rate equal to 0.5 to drop some neurons during training randomly. Furthermore, the Conv1 layer [53] consists of a 1 × 3 kernel size with 64 filters and a 1 × 1 stride. The 1D convolution operation applied on the input data *x*^*l*−1^ of the previous layer is defined in the following equation:

(14) $$\begin{array}{c} x^{l}=W^{l}\cdot x^{l-1}+b^{l}, \end{array}$$

The output is defined as *x*^*l*^ where *W*^*l*^ and *b*^*l*^ represent the weight matrix and the bias corresponding to the *l*-th layer, respectively. Meanwhile, two types of pooling were used, which are max-pooling and adaptive average pooling [54] with size 2 × 2.

As Figure 3 shows, the extract feature maps after the last pooling operation are inputted to a sequence of FC layers. The layers FC1, FC2, and FC3 were employed for feature extraction, whereas FC4 was used for the classification task. The FC4 used the Softmax function to output the probability of classifying a traffic sample to a specific type. As a regularization method, the CNN model uses batch normalization (BN) to normalize the input features fed to the FC4. The extracted feature vector from FC3 of each sample is of size 1 × 64. The extracted features are fed into the FS algorithm, which only selects the most relevant features to boost the overall performance of the intrusion detection task.

### 4.3. Third Phase: Feature Selection

During this phase, the proposed model selects the relevant features based on their quality. Thus, this process has a significant impact on IDS detection in IoT environments.

**Figure 4:**
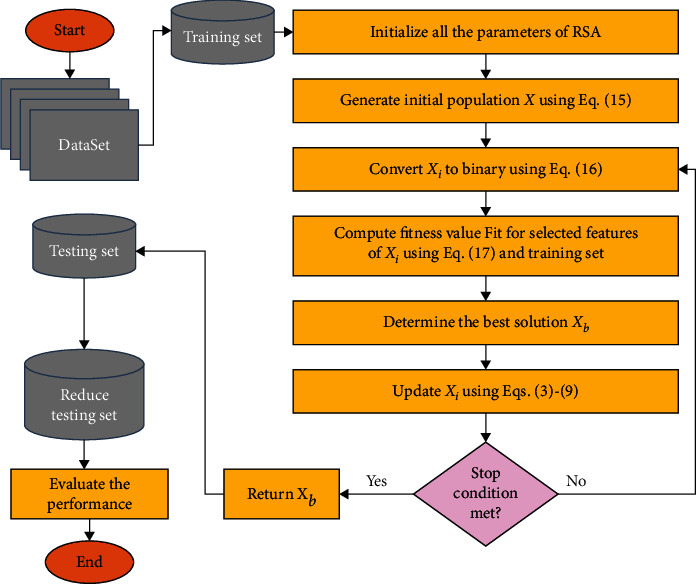
Steps of the RSA as an FS model for IoT security.

The proposed RSA as FS approach (see Figure 4) begins by initializing *X* population, with a number of agents represented by *N*. After that, it converts each agent into its binary version. More so, it reduces the number of features excluding those related to zeros from the binary version. Thereafter, the proposed RSA approach assesses the quality of the chosen features by computing the error classification according to the KNN classifier. Then, the best solutions (agents) are updated till reaching the optimal solutions.

#### 4.3.1. Create Population

The proposed RSA begins by dividing the given datasets into training and testing subsets, with 80% and 20%, respectively. After that, the following equation is applied to construct the initial values of population *X* with *N* agents:

(15) $$\begin{array}{c} X_{i}=\text{LB}+\text{rand}\left(1,D\right)\times \left(\text{UB}-\text{LB}\right), \end{array}$$

where *D* represents the dimension of each agent, which means the number of features. More so, rand(1, *D*) refers to a random vector, and LB and UB indicate the limits of the search space.

#### 4.3.2. Updating Population

In the updating phase, each *X*_*i*_ agent is converted into its Boolean version, as in the following equation:

(16) $$\begin{array}{c} {\text{BX}}_{ij}=\left\{\begin{array}{cc} 1, & \text{if}\;X_{ij}>0.5,\\ 0, & \text{otherwise.} \end{array}\right. \end{array}$$

Accordingly, feature numbers in the training set can be decreased by eliminating the features that belong to zeros. After that, the fitness value for each *X*_*i*_ agent is computed, as follows:

(17) $$\begin{array}{c} {\text{Fit}}_{i}=\left(1-\lambda \right)\times \left(\frac{\left|{\text{BX}}_{i}\right|}{D}\right)+\lambda \times \gamma_{i}, \end{array}$$

where *γ*_*i*_ refers to the classification error, which is computed utilizing the KNN depending on the training sets. More so, *λ* ∈ [0,1] represents random weights that are applied for balancing between classification error and the ratio of relevant features (|BX_*i*_|/*D*). To simplify this process, suppose *X*_*i*_=[0.72, 0.12, 0.09, 0.69, 0.21, 0.82, 0.75]. By applying (16), then BX_*i*_=[1,0,0,1,0,1,1]. Accordingly, the first, fourth, sixth, and seven features can be set as relevant features, where the training set can be decreased using them. Then, (17) is used to evaluate the quality of this section process.

The next stage is to obtain *X*_*b*_, which got the best fitness value Fit_*b*_. Thereafter, the *X*_*b*_ is used for updating the current agents using the operators of the RSA.

#### 4.3.3. Stop Learning Phase

During this phase, if the terminal criteria are not met, they will be checked. In this case, the updating process will be implemented again. Otherwise, *X*_*b*_ is considered as output, and it is applied to reduce the testing set that is used in the next phase.

### 4.4. Fourth Phase: Evaluation Performance

To evaluate the performance of the developed RSA, the best agent *X*_*b*_ is employed for ignoring, from the testing set, those features that correspond to zeros and are considered irrelevant. Then, compute the accuracy of the classification using several evaluation measures.  Algorithm 1 presents the full steps of the proposed RSA.

The complexity of the developed method RSA is *O*(RSA)=*O*(*N* × (*T* × *D*+1)).

## 5. Experimental Series and Results

This section presents the evaluation experiments of the developed IoT security approach and the evaluation process based on different evaluation metrics and real-world datasets and extensive comparisons to different methods in terms of features selection techniques.

### 5.1. Evaluation Measures

Several evaluation indicators are used to assess the quality of the proposed approach and all comparative methods.

We define those indicators according to the concept of the confusion matrix (see Table 1).

#### 5.1.1. Average Accuracy (AV_Acc_)

It refers to the rate of correct detection of intrusion. It can be calculated as

(18) $$\begin{array}{c} {\text{AV}}_{\text{Acc}}=\frac{1}{N_{r}}\sum_{k=1}^{N_{r}}{\text{Acc}}_{\text{Best}}^{k},\\ {\text{Acc}}_{\text{Best}}=\frac{\text{TP}+\text{TN}}{\text{TP}+\text{FN}+\text{FP}+\text{TN}}, \end{array}$$

where *N*_*r*_=30, which refers to the iteration number(number of runs).

#### 5.1.2. Average Recall (AV_Sens_)

This is also known as a true-positive rate (TPR), and it refers to the percentage of intrusion predicted positively. It is calculated as

(19) $$\begin{array}{c} {\text{AV}}_{\text{Sens}}=\frac{1}{N_{r}}\sum_{k=1}^{N_{r}}{\text{Sens}}_{\text{Best}}^{k},\\ {\text{Sens}}_{\text{Best}}=\frac{\text{TP}}{\text{TP}+\text{FN}}. \end{array}$$

#### 5.1.3. Average Precision (AV_Prec_)

It represents the percentage of TP cases of all positive cases. It can be computed as

(20) $$\begin{array}{c} {\text{AV}}_{\text{Prec}}=\frac{1}{N_{r}}\sum_{k=1}^{N_{r}}{\text{Prec}}_{\text{Best}}^{k},\\ {\text{Prec}}_{\text{Best}}=\frac{\text{TP}}{\text{FP}+\text{TP}}. \end{array}$$

#### 5.1.4. Performance Improvement Rate (PIR)

It is used to compute the rate of the improvement got by the developed method, and it is defined as

(21) $$\begin{array}{c} \text{PIR}=\frac{M_{\text{RSA}}-M_{\text{Alg}}}{M_{\text{RSA}}}\times 100, \end{array}$$

where *M*_RSA_ and *M*_Alg_ indicate the value of measure (i.e., precision, accuracy, recall, and F1-measure) of RSA and other algorithms, respectively.

### 5.2. Experiments Setup

The proposed CNN model in this study was trained for 100 epochs with early stopping using 2024 samples in each training batch. We save the best model during the training, resulting in a good performance on each dataset. The Adam [55] optimizer was used, where the learning rate is set to 0.005. The CNN model has been trained on a GPU of type Nvidia GTX 1080 and implemented using Pytorch framework1. The complexity of the CNN can be measured using the total updated parameters during the training, which is equal to 63,432. The proposed RSA was evaluated and compared to the following optimization algorithms: multiverse optimization algorithm (MVO) [56], whale optimization algorithm (WOA) [57], moth flame optimization (MFO) [58], grey wolf optimizer (GWO) [59], transient search optimization (TSO) [60], Bat (BAT) algorithm [61], and firefly algorithm (FFA) [62]. The parameters of each of these algorithms are set according to its implementation. However, the common parameters such as the number of iterations and agents are 50 and 20, respectively.

### 5.3. Dataset Description

To validate the proposed framework, we used KDDCup-99, NSL-KDD, CICIDS-2017, and BoT-IoT datasets. These datasets are the well-known datasets used to assess the IDS techniques, whereas the KDDCup-99 and NSL-KDD datasets share the exact source of data and the same intrusion type labels. Both KDDCup-99 and NSL-KDD were used to compare the proposed framework with other methods. Tables 2–4 list the datasets and the corresponding labels and samples distribution in training and testing sets.

The NSL-KDD dataset was built based on KDDCup-99, representing the refined version without duplicated network traffic samples. During the challenge on intrusion detection held by DARPA (defense advanced research projects agency) in 1998, the KDDCup-99 was created. The KDDCup-99 data were gathered from MIT Lincon laboratory experiments, where network traffic data were recorded during a period of 10 weeks. The setup used to experiment was around 1000 UNIX machines and 100 users. The collected network traffic data were around 5 million records stored in a raw transmission control protocol/Internet protocol (TCP/IP) dump format. Due to the enormous size of the dataset, the data collectors released a minor version representing only 10% of the total connection records consisting of 41 features for each record and the following types of attack: denial-of-service (DoS), probing, remote-to-user (R2L), and user-to-root (U2R). Meanwhile, the Bot-IoT dataset [63] consists of more than 72 million connection records gathered from many IoT devices. The dataset was collected by the Cyber Range Lab (at the UNSW Institute for Cyber Security) in Australia. We only used 5% of the entire dataset in our experiments, consisting of around 3.5 million records with ten features. The CICIDS-2017 [64] consists of 79 network flow features from gathered network traffic using the CICFlowMeter tool. The CICIDS-2017 datasets were collected by the CIC (Canadian Institute for Cybersecurity) to emulate real-world data (PCAPs). In addition, the collected connection records cover a variety of network protocols, including SSH, e-mail, HTTP, and FTP protocols generated by 25 users on machines with varying operating systems.

### 5.4. Results and Discussion

The results of the IoT security model based on the integration of the CNN and RSA compared with other models are given in this section. Tables 5 and 6 illustrate the average of each performance measure among the 25 independent number of runs for both binary and multiclass cases.

The analysis of the results in the multiclassification case can be noticed in the following points. The first point is that the efficiency of the developed RSA is better than the competition algorithms' overall performance measures during the learning stage among KDD99, NSL-KDD, and CIC2017. However, the performance of the RSA at BIoT achieved the second rank, following the MFO, which has better results. The second point that can be noticed is that the ability of RSA to detect the attack type using testing samples is higher than other methods when using the four dataset.

Furthermore, we can notice from the results of the algorithms in the case of the binary classification of the four datasets the high performance of the RSA either in the learning stage or evaluation stage. However, it can be noticed that high quality is achieved in the case of KDD99 and NSL-KDD. However, the result outcomes of the competitive methods are nearly the same in the other two datasets (i.e., BIoT and CIC2017), with little better performance for the developed method.

**Figure 5:**
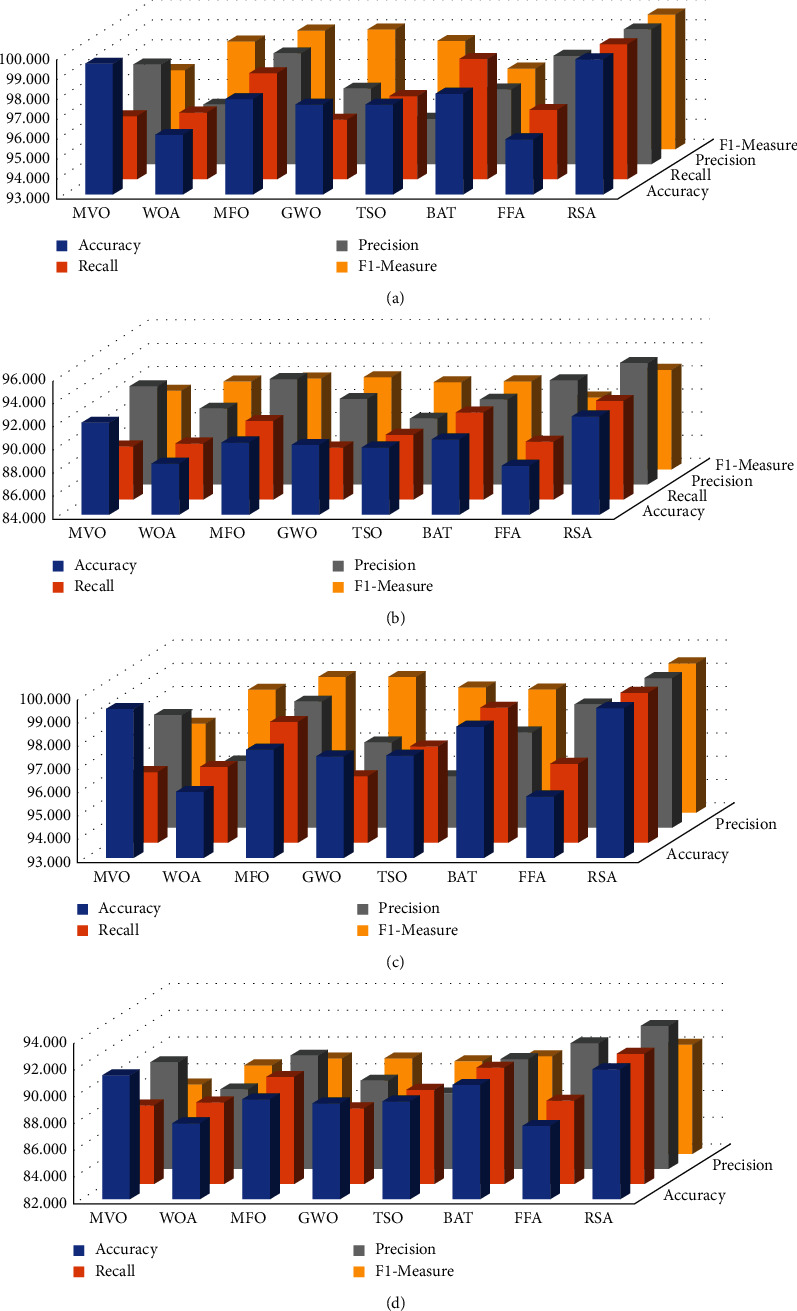
The average of all the tested sets the binary and multiclassification. (a) Binary, (b) binary, (c) multi, and (d) multi.

Moreover, Figure 5 depicts the average of each method among all the tested datasets in terms of each performance measure. It can be observed from this figure that the RSA has a high average overall performance metrics in the training and testing stages of the binary and multiclassification, followed by MVO in the multiclassification case, which provides better accuracy results than other algorithms. The BAT has a better recall value in the training and testing stages, and provides a better F1-measure value in the testing stage. Each of MFO and GWO, in the case of training, has higher precision and F1-measure value than other algorithms, whereas, in the case of the testing stage, FFA has a higher precision value than other methods. The same observation for MVO can be noticed in the case of binary classification. Each of MFO and GWO has better performance in terms of F1-measure and precision, respectively, in the training and testing stages. BAT provides better Recall value among the tested datasets in either the training or testing stages.

For further analysis of the obtained results, we used the Friedman test [65] to check whether the difference between the competition methods is significant or not. The Friedman test provides us with a mean rank for each method as given in Table 7. From these mean ranks, we can conclude that the mean rank of RSA is the highest in terms of performance measures in both classification scenarios (binary and multiclass), followed by MVO, FFA, MFO, and BAT, which has a high mean rank according to accuracy, precision, F1-measure, and recall, respectively.

From the previous results, it can be noticed the high ability of the developed method to improve the process of predicting the attack in the IoT environment. However, the developed method has some limitations, such as being time-consuming resulting from learning the model. However, this can be fixed by using transfer learning techniques.

## 6. Conclusion

This article presented a new method for intrusion detection systems (IDSs) of the Internet of things (IoT) and cloud environments. The main idea is to utilize the proliferation of deep learning and metaheuristic optimization algorithms to build robust feature extraction and selection techniques. First, a one-dimensional convolutional neural network (CNN) method is suggested to extract the relevant features. Second, the reptile search algorithm (RSA) is employed to select an optimal feature subset to reduce data dimensionality and boost classification accuracy. Several well-known and public datasets were used to assess the performance of the suggested techniques. More so, extensive experimental comparisons were carried out to confirm the quality of the RSA as a feature selection technique. The outcomes revealed that the RSA obtained better performance compared to several optimization approaches, such as PSO, FA, GWO, WOA, TSO, BAT, and MVO. It recorded over 99% for all training scenarios of all datasets. Also, it recorded high results in a testing scenario; for example, for multiclassification, the RSA obtained 92.040%, 89.684%, 89.985%, and 92.040%, of accuracy, precision, F1, and recall, respectively, for KDD99 datasets. Also, in the binary classification, the proposed method recorded high results; for example, it recorded 92.344%, 94.335%, 92.763%, and 92.344%, of accuracy and precision, F1, and recall, respectively, for KDD99 datasets in the testing scenario. For other datasets, the proposed RSA also recorded superior results in all evaluation tests using several classification indicators. We concluded that the applications of CNN with RSA have significant impacts on the IDS classification process. For future work, other issues could be addressed; for example, the convergence speed of the RSA needs to be improved. Thus, other artificial search mechanisms could be integrated with the RSA to tackle this problem. Also, in future work, we may consider applying the RSA for training deep learning models to boost the classification process for different applications, including IDS.

## Figures and Tables

**Algorithm 1:**
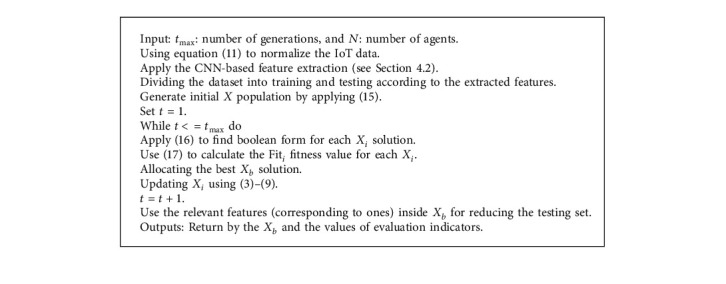
Developed IoT for feature selection.

**Table 1:** The confusion matrix. Note that “TP represents true positive, FN indicates false negative, false positive is represented by FP, and TN represents true negative.”

Actual label	Predicted label	
	Positive	Negative
Positive	TP	FN
Negative	FP	TN)

**Table 2:** Attack types in KDDCup-99 and NSL-KDD.

Dataset	Split	U2R	DoS	R2L	Probe	Normal
NSL-KDD	Train	52	45,927	995	11,656	67,343
	Test	67	7458	2887	2422	9710

KDDCup-99	Train	52	391,458	1126	4107	97,278
	Test	228	229,853	16,189	4166	60,593

**Table 3:** Attack types in Bot-IoT.

Bot-IoT	Split	DDoS	DoS	Reconnaissance	Theft	Normal
	Train	1,541,315	1320,148	72,919	65	370
	Test	385,309	330,112	18,163	14	107

**Table 4:** Attack types in CICIDS-2017.

CICIDS-2017	Split	DDoS	FTP-Patator/SSH-Patator	PortScan/Brute Force	Sql Injection/XSS	Benign
	Train	112,901	6997/5201	140,043/1329	19/575	72,7397
	Test	25,388	1574/1169	31,492/299	4/129	163,572

**Table 5:** Binary classification results of all algorithms.

		Training				Testing			
		Accuracy	Precision	F1-measure	Recall	Accuracy	Precision	F1-measure	Recall
KDD99	MVO	99.519	96.489	94.439	92.839	91.844	90.765	92.701	85.164
	WOA	92.278	92.418	97.308	93.128	84.608	86.699	92.705	85.458
	MFO	96.079	97.639	98.379	97.129	88.413	91.922	92.710	89.463
	GWO	95.518	94.068	98.488	92.388	87.860	88.357	92.716	84.730
	TSO	95.298	90.825	97.332	94.592	87.593	85.280	92.541	87.090
	BAT	94.992	92.922	91.782	98.662	87.384	87.280	92.751	91.055
	FFA	91.987	97.327	91.537	93.367	84.327	91.614	92.713	85.707
	RSA	**99.921**	**99.921**	**99.921**	**99.921**	**92.344**	**94.335**	**92.763**	**92.344**

NSL-KDD	MVO	99.197	96.167	94.117	92.517	76.466	79.835	71.059	69.786
	WOA	91.959	92.099	96.989	92.809	69.409	75.972	74.115	70.259
	MFO	95.760	97.320	98.060	96.810	73.187	81.176	75.162	74.237
	GWO	95.202	93.753	98.172	92.072	72.944	77.801	75.609	69.814
	TSO	95.091	90.681	97.091	94.571	72.078	73.656	73.786	71.558
	BAT	97.693	94.533	97.023	97.933	75.192	78.473	74.197	75.432
	FFA	91.673	97.013	91.223	93.053	69.218	80.944	68.451	70.598
	RSA	**99.233**	**99.235**	**99.233**	**99.233**	**77.814**	**83.830**	**77.545**	**77.814**

BIoT	MVO	99.990	99.959	99.939	99.923	99.989	99.958	99.937	99.922
	WOA	99.918	99.919	99.967	99.926	99.916	99.916	99.965	99.924
	MFO	99.956	99.971	99.978	99.966	99.954	99.969	99.976	99.964
	GWO	99.950	99.935	99.979	99.919	99.948	99.933	99.977	99.917
	TSO	99.949	99.905	99.969	99.944	99.947	99.903	99.967	99.942
	BAT	99.975	99.943	99.968	99.977	99.973	99.941	99.966	99.975
	FFA	99.915	99.968	99.910	99.928	99.913	99.966	99.908	99.927
	RSA	**99.994**	**99.994**	**99.994**	**99.994**	**99.993**	**99.992**	**99.992**	**99.993**

CIC2017	MVO	99.577	99.427	99.457	99.417	99.577	99.427	99.457	99.417
	WOA	99.730	99.537	99.470	99.531	99.737	99.537	99.497	99.737
	MFO	99.407	99.417	99.427	99.477	99.407	99.417	99.527	99.477
	GWO	99.417	99.477	99.427	99.607	99.417	99.477	99.427	99.607
	TSO	99.724	99.744	99.436	99.654	99.725	99.785	99.725	99.755
	BAT	99.537	99.667	99.472	99.647	99.537	99.667	99.487	99.687
	FFA	99.497	99.517	99.470	99.601	99.497	99.517	99.647	99.787
	RSA	**99.996**	**99.996**	**99.996**	**99.996**	**99.997**	**99.997**	**99.997**	**99.997**

**Table 6:** Mutliclassification results of all algorithms.

		Train				Test			
		Accuracy	Precision	F1-measure	Recall	Accuracy	Precision	F1-measure	Recall
KDD99	MVO	99.515	96.483	94.433	92.835	91.615	86.649	84.480	84.935
	WOA	92.275	92.414	97.304	93.126	84.375	82.501	87.351	85.225
	MFO	96.073	97.631	98.371	97.123	88.175	87.763	88.420	89.225
	GWO	95.513	94.062	98.482	92.383	87.618	84.131	88.533	84.488
	TSO	95.439	91.027	97.437	94.919	87.536	80.791	87.479	87.016
	BAT	98.007	94.847	97.337	98.247	90.347	89.134	**90.093**	90.587
	FFA	91.988	97.328	91.538	93.368	84.318	**91.609**	84.285	85.698
	RSA	**99.910**	99.909	99.906	**99.910**	**92.040**	89.684	89.985	92.040

NSL-KDD	MVO	99.182	96.145	94.093	92.502	75.224	75.200	66.098	68.544
	WOA	91.947	92.080	96.968	92.797	67.951	71.131	68.907	68.801
	MFO	95.745	97.297	98.035	96.795	71.626	76.122	69.844	72.676
	GWO	95.182	93.724	98.143	92.052	71.066	72.151	69.948	67.936
	TSO	95.078	90.657	97.067	94.558	71.330	71.298	69.697	70.810
	BAT	97.669	94.501	96.989	97.909	73.671	73.501	68.905	73.911
	FFA	91.660	96.991	91.201	93.040	67.437	75.873	62.944	68.817
	RSA	**99.201**	**99.158**	**99.148**	**99.201**	**76.107**	**82.171**	**71.731**	**76.107**

BIoT	MVO	99.468	99.468	99.468	99.468	**99.031**	99.000	98.980	98.964
	WOA	99.472	99.472	99.472	99.472	98.956	98.957	99.005	98.964
	MFO	**99.480**	**99.480**	**99.480**	**99.480**	98.998	99.013	99.020	99.009
	GWO	99.477	99.476	99.476	99.477	98.990	98.975	99.019	98.959
	TSO	99.460	99.459	99.459	99.460	98.986	98.941	99.005	98.981
	BAT	99.475	99.475	99.474	99.475	99.019	98.987	99.012	99.021
	FFA	99.479	99.478	99.478	99.479	98.954	99.007	98.949	98.968
	RSA	98.829	98.829	98.829	98.829	99.020	**99.098**	**99.070**	**99.038**

CIC2017	MVO	99.530	99.390	99.410	99.370	99.270	99.120	99.150	99.110
	WOA	99.690	99.490	99.450	99.690	99.430	99.240	99.190	99.430
	MFO	99.360	99.370	99.480	99.430	99.100	99.120	99.220	99.170
	GWO	99.370	99.430	99.380	99.560	99.110	99.180	99.120	99.300
	TSO	99.680	99.750	99.680	99.710	99.420	99.480	99.420	99.450
	BAT	99.490	99.630	99.440	99.640	99.230	99.360	99.180	99.380
	FFA	99.450	99.480	99.600	99.740	99.200	99.220	99.350	99.490
	RSA	**99.911**	**99.910**	**99.889**	**99.911**	**99.911**	**99.907**	**99.888**	**99.911**

**Table 7:** Results of the Friedman test.

	MVO	WOA	MFO	GWO	TSO	BAT	FFA	RSA
Multiclassification								
Accuracy	6.7500	3.2500	4	3.2500	4	5.5000	1.5000	7.7500
Precision	3.8750	2.5000	5.1250	3	2.7500	5	6	7.7500
F1-measure	2	3.6250	5.7500	5	4.8750	4.7500	2.2500	7.7500
Recall	1.8750	3.3750	5	1.5000	5.2500	6.2500	4.7500	8
Binary classification								
Accuracy	6.5000	3.2500	4.2500	3.7500	4	4.7500	1.5000	8
Precision	4.2500	2.7500	5.5000	3.2500	2.5000	4.2500	5.5000	8
F1-measure	2	3.5000	5.2500	5.2500	4	4.7500	3.2500	8
Recall	1.5000	3.5000	5	1.7500	5.2500	6.2500	4.7500	8

## Data Availability

The data used to support the findings of this study are available from the authors upon request.
